# End-of-Life Care and Nurse’s Roles

**DOI:** 10.5152/eurasianjmed.2022.22324

**Published:** 2022-12-01

**Authors:** Ulviye Aydan Nacak, Yasemin Erden

**Affiliations:** 1 Department of Nursing, Erzurum Teknik University Faculty of Health Sciences, , Erzurum, Turkey

**Keywords:** Nursing, nursing roles, palliative care, end-of-life care

## Abstract

Every individual who opens his/her eyes to life at birth closes eyes to death at the end of life. Every individual who is in the process of death wants to spend the last periods of his life as free from pain as possible, in a dignified and peaceful way. End-of-life care is provided by nurses, which aims to alleviate the suffering of individuals in the dying process and to provide patients with a good death experience. The continuation of treatment-oriented practices in the end-of-life period causes the inability to provide end-of-life care and patients suffer from unnecessary practices. Nurses who are in one-to-one contact with patients have important roles in making the decision to switch to end-of-life care and in providing end-of-life care to patients. In the present article, the concept of end-of-life care is examined, the difference between end-of-life care and palliative care, which are often confused, is discussed, and the purpose of end-of-life care and its importance for patients are emphasized. The roles and responsibilities of nurses in end-of-life care are also highlighted. It has been discussed that the roles of nurses are very important in the transition from treatment-oriented care to comfort-oriented end-of-life care, providing effective end-of-life care, and patients experiencing a good death with dignity.

**Main Points**

- End-of-life care is a holistic process that includes the needs of both patients and their families.
- In this process, all the needs of the patient and their families should be evaluated holistically.
- Nurses’ knowledge and skills are important in the care of individuals and families in the end-of-life period.
- By displaying professional roles and attitudes, nurses can ensure that the patient ends his life in a hopeful environment where he is honored, respected, and surrounded by loved ones with end-of-life care.

## Introduction

It is a fact that death, which is an inevitable reality, will be experienced by every individual. Every individual wants to complete the death process with dignity, free from pain, fulfilling their last wishes, and receiving the support of their loved ones.^1^ End-of-life care aims to relieve the pain of the individual in the death process and provide a dignified death experience from the moment when the curative treatment no longer brings any benefit.^2,3^ However, end-of-life care cannot be provided to patients because of the treatment practices that prolong life for a certain time and do not cure the patient, and patients suffer from unnecessary routine practices in the last stages of life.^4-6^ Patients who spend the last period of their lives in pain cannot say goodbye to their families, fulfill their last wishes, and maintain their autonomy.^1,7,8^ Patients prefer a few pain-free days, in other words, end-of-life care, instead of prolonging their lives with pain.^1^ Nurses are the primary providers of end-of-life care in their professional roles^9^ because nurses are the individuals who stay with the patients for the longest time and are in one-to-one contact with them, are the first to observe that the patient does not respond to the treatment, see the preferences of the patient and the family, bring together the whole healthcare team, family and patient, and interpret and convey what they see correctly in line with their professional knowledge.^10,11^ In this way, nurses can guide the transition from treatment-oriented to comfort-oriented care with their roles as care managers and decision-makers,^12^ protect the rights of patients in the death process with the role of patient advocacy,^9^ and provide the necessary care and help patients and their family with their roles as an end-of-life care practitioner. At this point, it is required that nurses understand end-of-life care, distinguish between the aims of end-of-life care and palliative care, know the difference between patient groups in the 2 types of care, and be aware of the importance of end-of-life care for patients and their nursing roles and responsibilities in the end-of-life period.

## End-of-Life Care

Life and death are mutually complementary concepts that cannot be considered separately. Death is as real as life and a part of human life. The last unpreventable and fateful act of humans is death. When it is accepted that death, which is an inevitable end, is very close for the patient,^13^ end-of-life care is provided to patients until the last days and hours of life.^14^ This care protects the dignity of people through words and behaviors, helps people cope with their physical limitations, and protects and honors dying individuals.^3^ The purpose of this care is not to prolong life but to prevent or alleviate pain as much as possible while respecting the dying individual’s wishes. The focus of end-of-life care is to increase the individuals’ quality of life and ensure that they experience an honorable and peaceful death.^2,6,15,16^

## Palliative, Hospice, and End-of-Life Care

Palliative care, hospice care, and end-of-life care are similar but not the same, often used interchangeably, and can be confused.^17^ Knowing and understanding the purposes of these types of care will make it easier to understand end-of-life care.

Palliative care is patient- and family-centered care optimizing the quality of life by anticipating, preventing, and treating the individuals’ suffering because of their diseases. This care includes evaluating the physical, intellectual, emotional, social, and spiritual needs throughout the disease and supporting the individuals’ autonomy, providing the necessary information support, and assisting in care and treatment choices. Palliative care is similar but not the same as end-of-life care. In palliative care, an individual with a life-threatening disease has a chance of recovery and the care provided with medical treatment is effective. Palliative care often focuses on the pain, symptoms, and stress of serious diseases in addition to curative care methods. Also, the individual provided with palliative care does not need to be in the end-of-life period, and every individual who has a serious disease can receive palliative care, regardless of the prognosis of the disease.^2^

Hospice care is an end-of-life care model provided in private residences or institutions. Hospice care, which is accepted as a comfort-oriented care model, provided a compassionate approach for individuals who face a serious or life-limiting disease or injury, is based on alleviating the pain and suffering as much as possible while providing moral and emotional support suitable for the individual’s particular needs and wishes. Support is also provided to individuals’ relatives. Curative treatment is ineffective in hospice care and the focus is on providing more care. In most cases, care is provided in the individuals’ private residences but can also be provided in independent hospice facilities, hospitals, nursing homes, or other long-term care facilities. This care is also provided for patients who have a terminal prognosis and a life expectancy of less than 6 months.^2^

End-of-life care and palliative care are 2 types of care that are similar but have different purposes. Although patients do not respond to treatment in end-of-life care, they can improve in palliative care. Although the purpose of end-of-life care is to relieve the patient’s pain during the death process and to ensure an honorable death, the purpose of palliative care is to provide symptom control. Hospice care is the care provided to patients by healthcare professionals, mostly in paid institutions. End-of-life care can be provided in the last step of palliative care or hospice institution within hospice care. However, end-of-life care is not a type of care that can be given only in palliative wards or in hospice institutions in the final stages of patients. It must be provided to every individual who needs end-of-life care during the death process, regardless of where they are (i.e., home, intensive care units, palliative ward of an institution or hospital, etc.) because every individual deserves to die with dignity and peace in the death process.

## The Importance of End-of-Life Care for Patients

Every individual knows that s/he will experience death one day. However, despite this, people are afraid of death and diseases that they think are deadly and want to run away from.^18,19^ Ignoring the relationship that exists between life and death, death has mostly been considered something frightening and isolated from life. People who had terminal illnesses were abandoned in hospitals and intensive care units, and death was considered the end of everything such individuals were left to their loneliness and fate.^20^ Patients who are diagnosed with terminal diseases and their families were distanced, their questions were left unanswered, and routine treatment practices took the place of care.^4,21^ But the dying patient must be cared for as a unique individual who deserves respect instead of a case in which unnecessary diagnosis or useless treatments are tried.

Optimum end-of-life care must be given, regardless of age, conditions, and medical diagnosis to ensure that the dying patient can lead a high-quality and dignified life until the last moment of life.^22^ End-of-life care is based on ensuring the control of patients’ pain and symptoms,^23^ as well as maintaining their optimum well-being, reducing physical dependence as much as possible, reducing their physical dependence, creating an environment where they can spend quality time with their families and loved ones, and supporting them in finishing their unfinished businesses.^24^ When end-of-life care is not provided, the patient is affected negatively in many ways. Failure in providing effective end-of-life care, and especially pain management, may cause the patient to feel hopeless and withdrawn from all aspects of life, physically weak, and unable to decide on the treatment and care because of the decreased understanding.^7^

End-of-life care aims to reduce the dying individuals’ pain as much as possible. When end-of-life care is not provided to individuals, their pain will increase and they will feel physically tired because routine treatment and diagnostic practices continue. This can lead to a decrease in the patient’s energy and clarity of thought. Individuals who have reduced clarity of thought cannot decide on their own care and treatment.^7^ Individuals who are not allowed to determine the end of their own life or who cannot make their own decisions have less self-esteem, but protecting and honoring their autonomy is one of the basic principles of end-of-life care.^25,26^ The inability of healthcare professionals to explain to patients and their families about life-sustaining treatments also causes the individuals’ will to be ignored, and therefore, they have to continue to receive treatment-oriented care and suffer, even if they do not want it. End-of-life patients prefer comfort-oriented care (end-of-life care), in other words, they want that their pain reduced and sleep problems to be eliminated, rather than treatment-oriented care. Patients also say that they want to quit their lives in an environment where their dignity is supported, their preferences are respected, they are approached with compassion and they feel valued, and they want to live 2 days without pain rather than 2 years of suffering.^1^ Supporting patients to make end-stage treatment and care decisions is important in protecting their dignity and autonomy.

Maintaining the individuals’ autonomy in end-of-life care is not only limited to their participation in their own care and treatment decisions but also includes supporting them in daily life activities, being able to actively prepare for death, contributing to other individuals with their experiences, and allowing them to say goodbye to their loved ones.^27-29^

End-of-life care is essential for patients to experience a well-deserved dignity and a peaceful death that is as pain-free as possible. Failure to provide effective end-of-life care deprives patients of a well-deserved and respectable death experience, increases their suffering, damages their autonomy, lowers their quality of life, damages dignity, and does not allow them to say goodbye to their loved ones for the last time.

End-of-life care is also a process that includes the family of the patient. One of the aims of end-of-life care is to maximize the quality of life of the patient's family. With the correct determination of the needs of the family of the dying individual and the planning and implementation of nursing interventions for these needs, holistic care will be provided to the dying patient.

## Roles of Nurses in End-of-Life Care

Nurses are at the forefront of the care provided to patients nearing the end of their lives and their families.^30-32^ Nurses have very important roles and responsibilities toward patients and their families in the care of dying patients.^30,33-35^ Nursing practices are very important in providing the care that dying patients need in their search for a comfortable and dignified death.^10^ Nurses also have a unique place in the transition and provision of end-of-life care because nurses take care of patients and follow them, and they can observe that the patient does not respond to the treatment applied, but the patient suffers from pain as a result of the interventions. With these observations and knowledge, the nurse is the first person to realize that the patient needs end-of-life care. Nurses can transfer their observations and knowledge to other healthcare professionals and, together with the healthcare team, take an active part in the decision-making processes that reflect the patients’ physiological realities, preferences, and what can and cannot be done clinically, with their nursing roles, enabling the transition to end-of-life care.^36^

Nurses’ responsibilities and what they can do in the transition to end-of-life care in line with their nursing roles are as follows. In line with their manager role, nurses must cooperate with the family and health team to determine and verify the best treatment and care.^11,37^ However, nurses must also be able to take part in the caregiving process as the manager of the care by using their knowledge. As the manager of the care provided, the nurse must be able to take an active role in ensuring that the patient receives comfort-oriented care instead of treatment-oriented care, in other words, transition to end-of-life care.^12^ In line with their decision-making role, nurses must bring together all the people and stakeholders in the process of making the end-of-life care decision, share information about the patient and family, and ensure their comfort.^11^ The nurse must take part in the decision-making process of transitioning to end-of-life care by helping patients and families understand what they feel and by questioning the treatment decisions that may cause the suffering of patients, by considering their rights, interests, and preferences.^11^ As a result, in the process of transitioning to end-of-life care and making its decision, nurses can direct the care in line with their role as the managers of the care provided, enabling the patient and family to choose their own care and make decisions in line with their decision-making roles, and defend the rights of the dying patient with the role of patient advocacy. When it is decided to switch to end-of-life care, nurses provide comprehensive, honorable, and compassionate end-of-life care to patients in line with their roles as a practitioner^1,38^ and support patient relatives.^23^ In this process, nurses must ensure optimum pain management of the patient with end-of-life care^39^ and must protect patients and their families in terms of spiritual aspect and knowledge.^40,41^ Nurses must be in constant cooperation with other members of the healthcare team and the families of patients.^42,43^

By adopting a professional role and attitude, nurses can ensure that the patients end their lives in a hopeful environment where they are peaceful, respected, and surrounded by loved ones^44^ with end-of-life care.^45^ For this purpose, regardless of the time and place of death, nurses can create an environment that can bring the loved ones of patients together.^46^ Also, even in the absence of the family of the patient, the nurse must be a force that protects the patient during the death process and does not prolong or shorten the process but facilitates the situation for the patient/alleviates his/her suffering,^47^ and must not let him/her die alone.^10^ In line with all their roles, nurses must be able to ensure that the patient is actively prepared for the death process and has a well-respected death experience.^48-53^

## Conclusion and Recommendations

The care requirements and priorities of individuals at the last moment of their lives differ, as is the case in life. With end-of-life care, the purpose is to support the vital functions of the patient and spend as much as possible pain-free time before death with relatives in a dignified manner. Although the nurses who accompany and constantly care for patients in their last moments of life often witness their death, each patient and death is unique. Nurses have important roles in supporting patients and families in the transition from treatment-oriented care to effective end-of-life care. All practices that nurses will do in their roles in end-of-life care are important in ensuring that the patient experiences an honorable and good death. In the training to be provided to nursing students and nurses working in hospitals, it is recommended to emphasize the importance of nurses in providing effective end-of-life care for patients and the role of nurses in end-of-life care.
